# Community assembly following disturbance in batch anaerobic digesters displays highly reproducible secondary succession and a shifting stochastic-deterministic balance

**DOI:** 10.3389/frmbi.2025.1707779

**Published:** 2026-01-27

**Authors:** Flor de María Guerrero-Toledo, Teodoro Espinosa-Solares, Guadalupe Hernández-Eugenio, David H. Huber

**Affiliations:** 1 Departmento de Ingenierίa Agroindustrial, Universidad Autónoma Chapingo, Texcoco, Mexico; 2 Department of Biology, West Virginia State University, Institute, WV, United States; 3 Gus R. Douglass Institute, West Virginia State University, Institute, WV, United States

**Keywords:** community, anaerobic digestion, deterministic, microbiome, secondary succession, stochastic

## Abstract

The great diversity of anaerobic digestion (AD) microbiomes indicates high redundancy and flexibility in the assembly of the community. Moreover, AD microbiomes are frequently subjected to disturbances during start-up and operation that require (re)assembly. We tested the reproducibility of secondary succession and AD community assembly mechanisms using a pre-assembled microbiome that was subjected to intense disturbances. Microbiome diversity and functions were followed in replicate mesophilic batch digesters initiated with multiple stressors, including high feed-to-inoculum ratio and many foreign species. Three 10 L batch digesters were derived from a single long-term CSTR digester pre-adapted to poultry litter feedstock and operated in parallel. Physicochemical parameters (methane, acetate, propionate, butyrate, pH, N-NH3, COD) were measured. Metagenome samples were used to assess diversity and functions. Three performance phases were found along the successional gradient: (1) methane inhibition, (2) high methane production, and (3) low methane plateau. The inventory of species (>1600) remained nearly the same, however the relative abundance of species, families, and functions changed during each successional stage. Syntrophic bacteria peaked in abundance during the mid-succession, high methane stage. Succession of overall KEGG functions was highly similar although species and carbohydrate functions diverged during late succession, suggesting diversity of niche partitioning during degradation of recalcitrant organic matter. We estimated the relative contributions of stochastic and deterministic processes and found a shift in the balance during succession. Early succession was not dominated by either dispersal or selection while late succession was dominated by variable selection. In conclusion, methane production recovered following severe (non-lethal) disturbance in a pre-adapted digester microbiome through a reproducible community assembly pathway that shifted toward deterministic, variable selection over time.

## 1 Introduction

Understanding the drivers and controls of microbial community assembly has become a major theme in microbial ecology (Zhou and Ning, 2017). This is particularly important for engineered microbiomes where the desired functions of the microbiome depend on community structure. Community assembly is the ecological process where microbial species colonize and interact to form local communities through immigration from a regional species pool (Zhou and Ning, 2017). Community assembly can occur in uncolonized environments or following disturbance of an established microbiome. Both contexts represent ecological succession and are found in natural and engineered microbiomes, such as the human gut (Guittar et al., 2019) and waste water treatment plants (de Celis et al., 2022). Originally defined for plant ecology, primary succession occurs in uncolonized environments while secondary succession involves the (re)assembly of communities following substantial disturbances (Prach and Walker, 2011). Conceptually, both types of succession can be applied to industrial microbiomes (Fierer et al., 2010). However, the multitude of bioreactor designs, variations of substrates and products, experimental designs, and different types of stressors, makes studying assembly mechanisms challenging. Because of the importance of controlling assembly in engineered microbiomes, the drivers and controls of succession need to be further understood.

The pathways for microbial community assembly during succession involve both stochastic and deterministic mechanisms (Zhou and Ning, 2017). Stochastic processes are those involving ecological drift (random changes in population abundance) and dispersal. Dispersal can occur at a high rate which can homogenize community composition or low rate which allows for greater influence by drift, and greater community divergence. Deterministic processes, in contrast, are niche-based and emphasize the functional uniqueness of species, including metabolic capacity and interactions (Zhou and Ning, 2017). Selection is the mechanism whereby deterministic processes, coupled to environmental conditions, shape community structure.

The balance between stochastic and deterministic processes can change during community assembly depending on the degree of selection or dispersal, and environmental variability. Four ecological processes reflect shifts in this balance: homogeneous selection, variable selection, homogenizing dispersal, and dispersal limitation (Stegen et al., 2015). Homogeneous selection occurs in environments that are spatially uniform, leading to more similar communities, whereas variable selection occurs in heterogeneous environments that provide greater niche diversity and increased divergence among communities. Homogenizing dispersal occurs when high dispersal rates supersede selection, and interacting communities develop similar compositions. Dispersal limitation occurs when movement between communities is at a low level and selection is also weak. In this case, drift becomes the primary process, leading to divergence of communities. Finally, it may happen that neither dispersal nor selection dominate community assembly; these situations were designated as undominated by Stegen et al., (2015).

Secondary succession occurs following disturbances caused by biotic or abiotic events that cull populations, disrupt community functions, and alter habitats (Shade et al., 2012). Disturbances can be due to stochastic historical events (i.e., contingency) that alter the stochastic/deterministic balance as well as environmental context for community assembly (Zhou et al., 2014). Disturbances can therefore propel succession down multiple pathways that differ in species and functions. Indeed, multiple disturbances may act synergistically resulting in unexpected effects on assembly (Houseman et al., 2008), leading to the development of alternative states where final community compositions are distinct (Fukami and Nakajima, 2011; Fukami, 2015). In engineered microbiomes, the pathways of succession have been shown to be affected by inoculum diversity (De Vrieze et al., 2015; Perrotta et al., 2017), stressors (Regueiro et al., 2015), and stochastic processes (Zhou et al., 2013). This is important because bioreactors are often operated for the products produced by the transient state of the system, rather than final state where metabolic processes change dramatically. Type of disturbance and operational differences, though, make it unclear how reproducible successional trajectories actually are, and difficult to compare diverse systems.

Anaerobic digestion (AD) is a waste treatment process for highly concentrated organic wastes such as livestock manure, producing bioenergy as a byproduct. AD relies on the assembly of a community of bacteria and archaea that requires considerable interspecies cooperation (syntrophy) to convert heterogeneous organic molecules into methane through a multi-trophic level food web. Succession can occur during both digester start-up and operation. Start-up may involve a severe disturbance of a community when inoculum is taken from an established digester. In this case, the substrate to inoculum (S/I) ratio is particularly important for successful start-up and S/I ratios that are too high can lead to acidification and assembly of non-methanogenic communities (Li et al., 2022). Digesters also receive waste feedstock that contains thousands of foreign species that could possibly disrupt community structure, particularly following severe disturbances (Amor et al., 2020; Huber et al., 2020). Nevertheless, long-term operation of digesters with consistent feedstock selects persistent species indicating niche specialization, competitive exclusion, and stability (Werner et al., 2011).

Digesters are typically designed as either flow-through (steady state) or batch systems. Batch digesters represent an interesting case of ecological succession in a closed system. These communities rely on endogenous carbon and energy sources and experience directional change as resources are depleted and metabolites accumulate. However, the primary drivers of succession are not clear. The liquid environment might be expected to mitigate the role of dispersal in community assembly although decomposition of complex particulate organic matter over time could also increase environmental heterogeneity (Allison, 2012; Enke et al., 2018). These factors could shift the balance between stochastic and deterministic processes. In addition, microbial inoculum could be taken from a pre-adapted digester where species sorting and niche partitioning already occurred. Therefore, batch digesters are excellent systems for studying community assembly during secondary succession in bioreactors. Batch digesters are also used in many applications such as Biochemical Methane Potential (BMP) tests (Holliger et al., 2020).

Our objectives were to evaluate the repeatability of community assembly processes in batch mode digesters following disturbances that could produce alternative successional states. If deterministic processes are dominant during succession, it is expected that replicate communities will have highly similar structures due to prior environmental filtering of ecological specialists. However, stochastic processes and historical contingency could lead to alternative successional pathways. Metagenomics was used to investigate whether both species and functional diversity were affected by deterministic or stochastic processes. The start-up of replicate reactors used inoculum from a pre-adapted microbiome that could potentially drive deterministic assembly, but also a very high S/I ratio using animal manure feedstock containing a large reservoir of foreign microbes that could drive stochastic assembly.

## 2 Materials and methods

### 2.1 Experimental design

The experiment was designed to test the repeatability of successional pathways and investigate community assembly mechanisms using three replicate batch digesters started with the same pre-adapted microbiome as well as the same feedstock. The inoculum was taken from a 10 L CSTR (continuous stir tank reactor) digester that had been operated long-term with poultry litter. The inoculum was divided equally into three 10 L batch digesters with the same feedstock. The feedstock to inoculum (v/v) ratio was exceptionally high (19:1); far higher than recommended (2-4:1) for digester start-up (Li et al., 2022). The opportunity for stochastic turnover in the community was therefore increased by both feedstock microbial diversity and disturbance due to high dilution. In addition, the use of batch digesters allows a temporal resource gradient to form which facilitates the observation of successional stages and potential shifts in stochastic/deterministic processes.

Three reactor vessels (D1, D2, D3) with 13 L capacity and 10 L working volume were operated for 191 days at 37 ± 2 °C. The feedstock was poultry litter slurry with 3% total solids (TS) from a commercial farm in Tepetlaoxtoc, Mexico. The slurry was made the same day the experiment began by adding water to dry feedstock and filtering to remove large particles. Poultry litter consists of animal excrement, bedding material, and feed residue. The properties of the feedstock were previously reported (Meneses-Reyes et al., 2017). The poultry litter contained TS (66.28%), VS/TS (69.61%), protein (24.70%), ash (20.13%), carbohydrates (19.654%), crude fiber (16.25%), and C:N ratio (8.15) (Meneses-Reyes et al., 2017). The inoculum was 5% (v/v) which came from a CSTR digester adapted to chicken litter for 1283 days. When the inoculum was taken, the parent digester was under steady state conditions (HRT 30 d, 37.6 °C) with pH 7.6, 49.0% methane, and acetate and propionate levels of 1,077 and 682 mg L^-1^, respectively. Mixing was done once per day by hand rotation.

### 2.2 Analytical methods

Chemical oxygen demand (COD) was determined using standard methods (APHA standard methods for the examination of water and wastewater, 2005); ammonia nitrogen was determined with HACH Method 10031. Profiles of volatile fatty acids (VFA) were determined as described by (Meneses-Reyes et al., 2018) using gas chromatography (GC) (Claurus 500, Perkin Elmer) equipped with a flame ionization detector and a capillary column Elite-FFAP (length 30 m, diameter 0.32 mm). VFAs were determined using samples of effluent acidified to pH 3 with HCl and centrifuged at 14,500 rpm for 10 min. The supernatant was injected with an auto-sampler using 5 µL volume. Operational conditions were: gas flow 5 mL min^-1^ at 10.6 psi, 150 °C injection port, 100 °C oven for 8 min with ramp of 160 °C for 8.5 min, 250 °C detector, and retention time of 16.5 min. Helium was the carrier gas. Biogas production was quantified by the displacement method in saline water 10% (v/v) with a flowmeter that uses volume shift and a digital counter (LA8N-BN, Autonics). Methane analysis followed (Guerrero-Toledo et al., 2019) using a GC (Claurus 500, Perkin Elmer). 10 µL biogas samples were injected into the GC with conditions: flow 14 mL min^-1^ at 14 psi, 100 °C injection port, 70 °C oven, 100 °C detector, retention time 4 min, carrier gas helium. Percentage of methane was obtained using a calibration curve with pure methane. Biochemical Methane Potential (BMP) was calculated following (Meneses-Reyes et al., 2018).

### 2.3 Metagenomic analysis and statistics

Cell samples were taken three times corresponding to performance phases: beginning (P1), middle (P2), and end-point (P3). To extract cell samples, 50 mL of sludge was pelleted with centrifugation (6700 rpm) and stored at -80 °C. Genomic DNA was extracted using PowerSoil^®^ DNA Kit (Qiagen). Genomic DNA was stored at -80 °C before sequencing. Nine genomic DNA samples were sent to Admera Health (New Jersey) for library construction and sequencing. Library preparation was performed using Nextera XT. Library quality and quantity were assessed with Qubit 2.0 DNA HS Assay (ThermoFisher), Tapestation High Sensitivity D1000 Assay (Agilent Technologies, USA), and QuantStudio ^®^ 5 System (Applied Biosystems). Genomic DNA libraries were sequenced with the Illumina Hiseq 2x150bp format. Sequences were submitted to the National Center for Biotechnology Information Sequence Read Archive under BioProject PRJNA1327443. Sequence quality control used FastQC v.0.11.5. Primers and chimeras were removed using Trim Galore (Martin, 2011) before merging the paired-end sequences using FLASH (Magoč and Salzberg, 2011). All sequences (84.92 Gb) were also deposited in MG-RAST database (Wilke et al., 2015). MG-RAST version 4.0.3 was used for phylogenetic and functional assessments using default parameters (Wilke et al., 2015). Taxon abundance was normalized by dividing by the total number of hits in each metagenome. STAMP version 2.1.3 and R CRAN packages on R Studio and JMP were used for statistical analysis of metagenomic data. Hellinger-transformed data were used. Benjamini-Hochberg FDR correction was used for controlling false discovery rate instead of the familywise error. Scatter plots, hierarchal clustering and Principal Coordinates Analysis (PCoA) were used to evaluate differences in community dynamics and successional trajectories. Physico-chemical parameters (propionate, acetate, butyrate, pH, ammonia nitrogen [N-NH_3_], chemical oxygen demand [COD], and methane) were evaluated across three operational phases (P1, P2, and P3) using one-way Analysis of Variance (ANOVA). Additionally, Tukey’s Honestly Significant Difference (Tukey HSD) test was used for *post-hoc* comparisons. Model assumptions were evaluated using residual Q-Q plots and the Shapiro-Wilk test for normality and Levene’s test for homogeneity of variances.

Whether stochastic or deterministic processes were present as community assembly occurred was evaluated using the beta Nearest Taxon Index (βNTI) method (Stegen et al., 2012; Stegen et al., 2013). For this analysis, metagenomic taxonomic profiling was performed with MetaPhlAn 4.0 (Blanco-Míguez et al., 2023) using species-level marker genes. The resulting relative-abundance table was used as the community matrix for ecological distance calculations. A phylogenetic tree corresponding to the detected taxa was used to compute pairwise phylogenetic distances using cophenetic branch lengths with *phyloseq* function. Taxa absent from the tree or without a matching tip label were removed to ensure consistent taxon sets between the community matrix and the phylogeny. Observed abundance-weighted beta Mean Nearest Taxon Distance (βMNTD) was calculated for each pair of phases using the comdistnt() function in R (Lozupone and Knight, 2005), which quantifies the mean nearest phylogenetic neighbor distance between two communities. This provided a phase × phase matrix describing the observed phylogenetic turnover. Raup-Crick Bray-Curtis (RCbray) analysis was used to partition non-selection processes (drift vs dispersal) using the *vegan* package (Chase et al., 2011).

## 3 Results

### 3.1 Parallel performance of replicate digesters along a successional gradient

**Figure 1:**
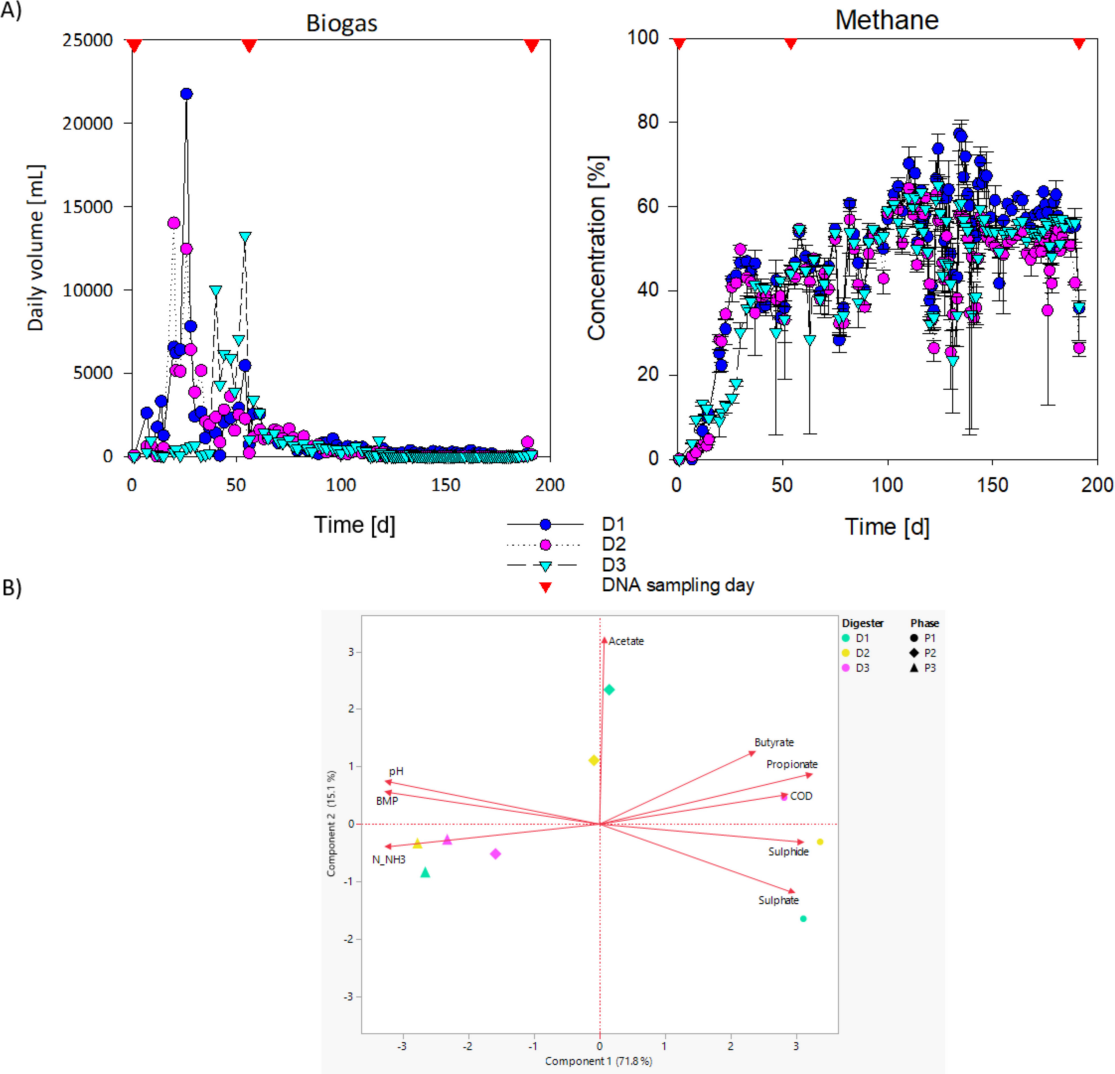
**(A)** Biogas and methane production during the three performance phases for each digester (D1, D2, D3). Time points for microbial diversity sampling are indicated by red arrows. **(B)** PCA analysis of digester performance variables for each successional stage (phase).

Significant differences were observed among operational phases for all measured parameters (methane, propionate, butyrate, acetate, pH, N-NH_3_, COD) (p < 0.05) indicating distinct process conditions and microbial activity in each successional stage (**Table 1**, **Table 2**). Parameters were measured at the same time points as biogas and microbiome sampling. Therefore, three operational phases were defined and associated with stages of community succession. We refer to these phases as: P1, start-up and methane inhibition; P2, maximum methane production; and P3, low methane plateau. Methane concentrations were significantly greater in P2 and P3 than in P1 (p < 0.01), consistent with increased methanogenic activity. During P1, biogas and methane were quite low indicating that dilution with poultry litter feedstock caused inhibition (**Figure 1A**). During P2, total biogas peaked on day 26 (D1), day 20 (D2), but much later in digester D3 (day 54). As biogas peaked, methane percentage reached 73.2% (D1), 65.5% (D2) and 63.7% (D3). In P3, methane percentage decreased to 31.4% (D1), 25.2% (D2), and 34.8% (D3). COD exhibited a clearly decreasing trend (P1 > P2 > P3, p < 0.001), reflecting progressive organic matter degradation. By P3, COD decreased by 86% in D1, 83% in D2 and 80% in D3 (**Table 1**). Propionate concentrations were highest in P1, and decreased sharply in P3 relative to P1 and P2 (p < 0.001) indicating enhanced syntrophic oxidation and improved methanogenic conditions (Yidan, 2023). Acetate showed its highest values in P2 (p = 0.018). Butyrate was significantly lower in P3 than in P2 (p = 0.048). pH increased progressively from P1 to P3 (all pairwise comparisons, p < 0.01), coinciding with the consumption of volatile fatty acids and increased ammonia release from protein degradation (Chen et al., 2008). Ammonia (N-NH_3_) concentrations were significantly higher in P3 compared to P1 (p = 0.003), indicating enhanced deamination activity in later phases. BMP was most similar in D1 and D2. By phase 3, BMP for D3 was 20-29% lower than the other digesters. The progression of metabolic changes was also evaluated with PCA using these performance variables. PCA showed that the phases grouped separately except P2 of D3. PCA component 1 explained 75.2% of the variation while component 2 explained 14.9% (**Figure 1**). Together, these results indicate distinct metabolic regimes for each operational phase.

**Table 1:** Changes in physicochemical parameters of three digesters during the experiment.

Parameter	D1P1*	D1P2	D1P3	D2P1	D2P2	D2P3	D3P1	D3P2	D3P3
Days	1-7	8-130	131-191	1-7	8-110	111-191	1-35	8-115	116-191
Propionate [mg L^-1^]	826.60	739.78	18.49	922.86	494.45	16.00	860.45	32.44	17.33
Acetate [mg L^-1^]	105.99	1115.62	65.78	233.48	685.91	312.87	349.16	185.83	311.68
Butyrate [mg L^-1^]	0.00	33.57	0.00	84.35	31.25	0.00	93.02	0.00	12.84
pH	6.82	7.77	8.05	6.86	7.79	8.01	6.84	7.74	7.94
N-NH_3_ [mg L^-1^]	483.80	789.40	1107.25	479.75	844.90	1302.35	501.2	1217.0	1146.05
COD [mg L^-1^]	27595.16	12392.50	3959.78	25457.29	11204.79	4316.09	26526.22	3484.69	5503.80
BMP	0	142.69	216.63	0	139.95	191.52	0	135.74	155.49

* D1, D2, D3 refers to digesters. P1, P2, P3 refers to performance phases. BMP (Biochemical Methane Potential).

**Table 2:** Summary of one-way ANOVA results for each physico-chemical parameter tested across digester phases (P1, P2, P3).

Parameter	F-statistic	p-value
Propionate [mgL-1]	45.31	0.00006
Acetate [mgL-1]	7.51	0.011
Butyrate [mgL-1]	5.72	0.022
pH	204.2	< 0.00001
N-NH_3_ [mgL-1]	15.87	0.0018
COD [mgL-1]	52.44	0.00004
Methane [%]	17.38	0.0014

### 3.2 Succession of community structure in replicate digesters

We compared the replicate digester communities to address four questions: Do performance phases correspond to community successional stages? How reproducible are the successional stages between digesters? Do both species and functions display similar trajectories? Are stochastic or deterministic processes dominant during secondary succession in batch digesters? Nine metagenomes were compared representing three performance phases in each digester. Both abundance of taxa and functions were evaluated. Based on classifiable sequences, the relative abundance of Bacteria ranged from 96.1 to 99.01%, and Archaea from 0.5 to 3.38%. Based on MG-RAST, the total taxonomic inventory consisted of 33 phyla, 64 classes, 129 orders, 273 families, 661 genera, and 1622 species. Total identifiable species in each digester was nearly identical: 1602 in D1, 1611 in D2, and 1601 in D3. The majority of species (95%) were shared in every sample.

**Figure 2:**
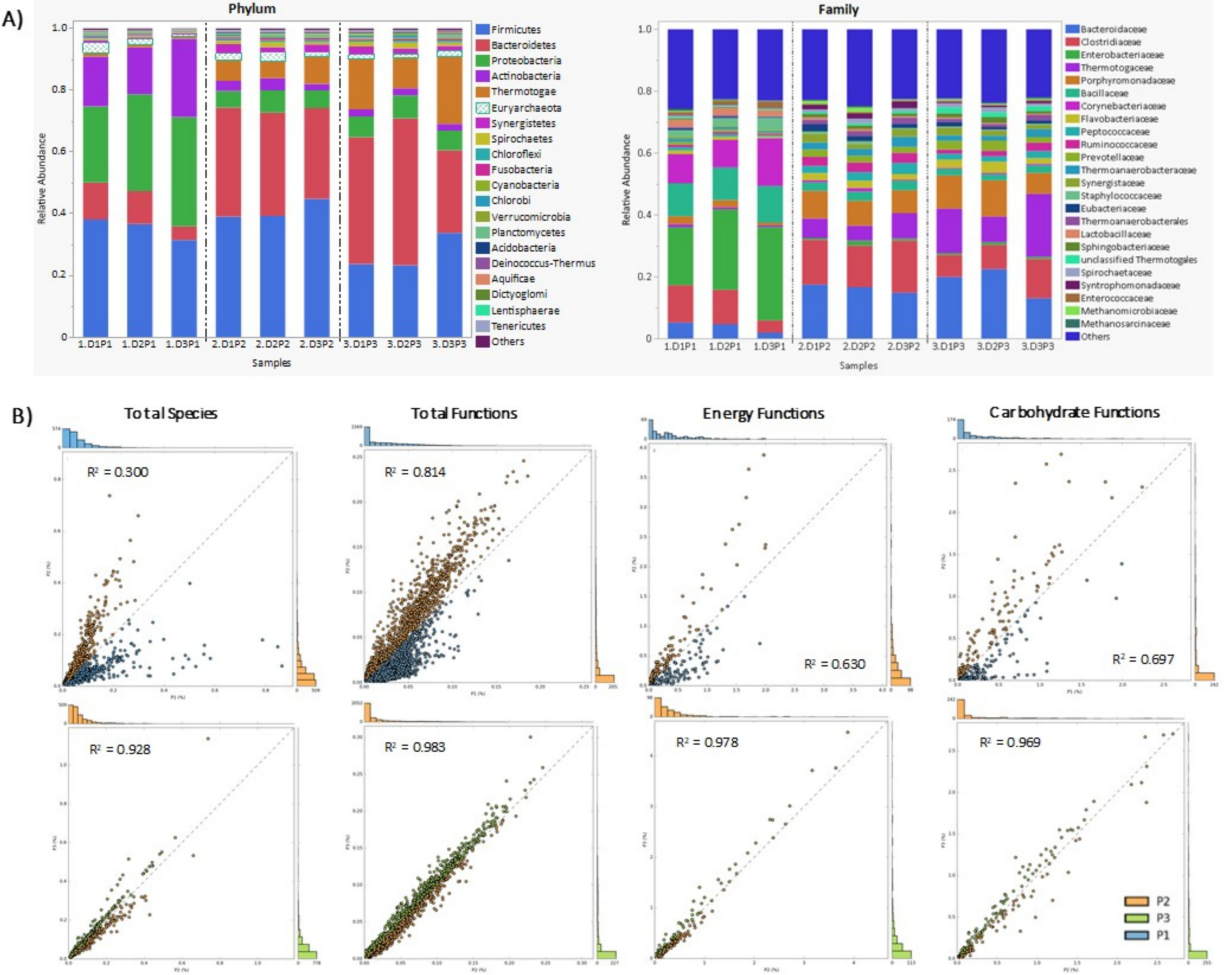
Demonstration that digester performance phases represent ecological succession of community structure. **(A)** Bar graphs showing alpha diversity of the most abundant phyla and families of Bacteria and Archaea during each digester phase. **(B)** Scatter plots to evaluate changes in the abundance of total species and functions between digester phases (P1, P2, P3). Functions are analyzed in terms of total KEGG functions, energy functions, and carbohydrate functions. For each pair of graphs, the top plot compares P1 and P2, and the bottom plot compares P2 and P3.

Overall similarity of digesters was evaluated with multivariate analysis, diversity indices, and correlation analysis. Digester identity showed no detectable effect on community structure with ANOSIM (R = –0.1605, p = 0.7565) and PERMANOVA (F = 0.188, p = 0.8177). The digesters were also compared in terms of Shannon, Simpson, and Fisher’s alpha indices (**Table 3**). Only Fisher’s alpha had a significant P-value (<0.0005). Fisher’s alpha is sensitive to rare species and high diversity. Using correlation analysis of Bacteria and Archaea separately, all three digesters were quite similar although D3 was somewhat less (**Table 4**; **Supplementary Figure 1**). For Bacteria, correlations were: D1/D2, R^2^ = 0.977; D1/D3 and D2/D3, R^2^ = 0.943). For Archaea, correlations were: D1/D2, R^2^ = 0.983; D1/D3, R^2^ = 0.941; D2/D3, and R^2^ = 0.945 (**Table 4**). The overall similarity of total functions between digesters was also very high: D1/D2, R^2^ = 0.994; D1/D3, R^2^ = 0.988; and D2/D3, R^2^ = 0.985. The dominant phyla in P1 were Firmicutes, Proteobacteria, and Actinobacteria; dominance shifted during P2 and P3 to Bacteroidetes, Firmicutes, and Thermotogae (**Figure 2A**). Abundance of Euryarchaeota varied the most among digesters during P1 (3.3% D1, 1.8% D2, 0.51% D3), but converged by P3 (1.4-1.9%).

**Table 3:** Diversity indices comparing community similarity for the three digesters as replicates.

Diversity index	F value	P-value
Shannon	2.282	0.183
Simpson	2.183	0.194
Inv Simpson	1.445	0.307
Fisher	35	< 0.0005

**Table 4:** Correlation analysis (R^2^) to evaluate the similarity of the three digesters as replicates.

Digester comparisons	Bacteria species	Archaea species	Total KEGG sunctions
D1/D2	0.977	0.983	0.994
D1/D3	0.943	0.941	0.988
D2/D3	0.943	0.945	0.985

**Figure 3:**
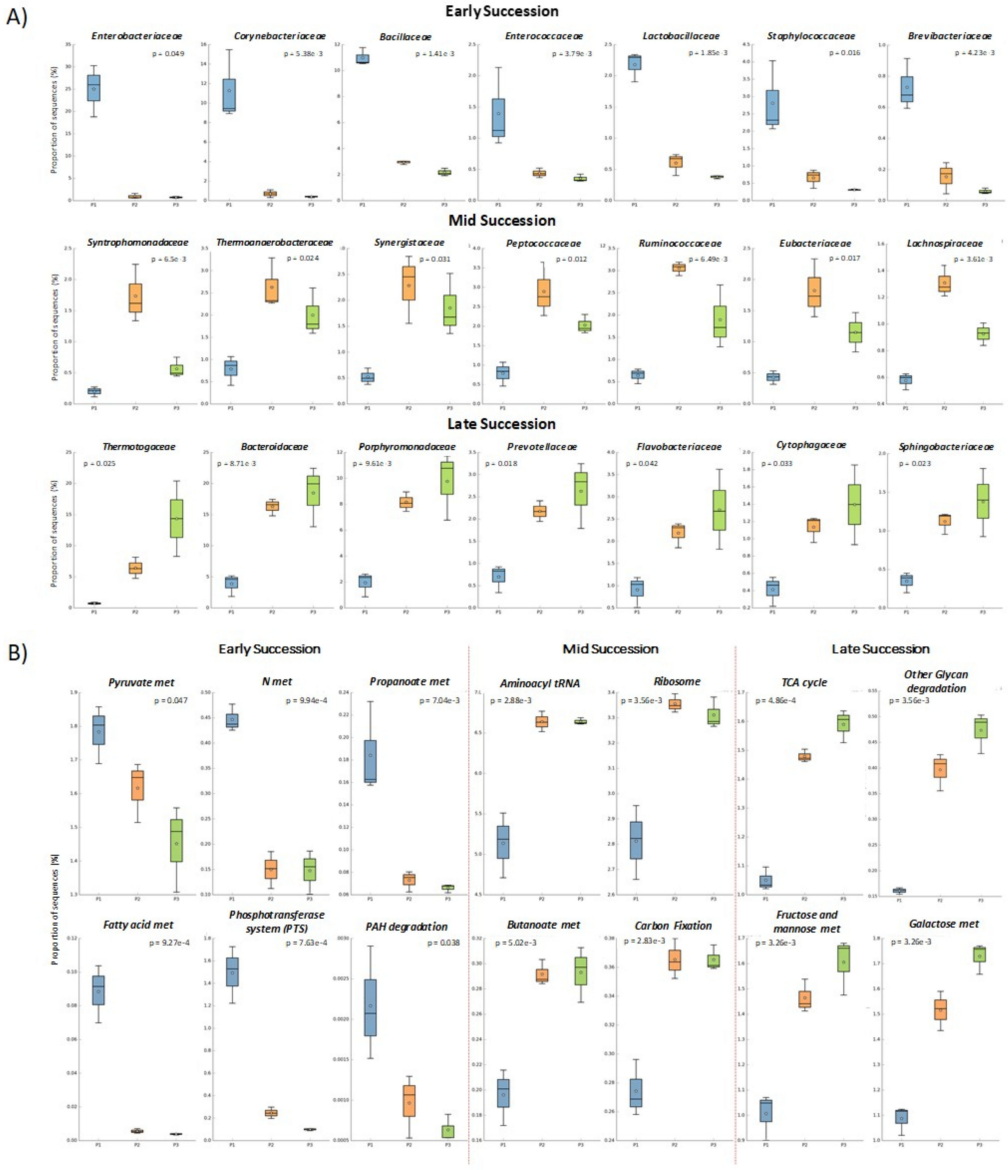
Box plots showing that particular bacterial families **(A)**, and specific functions **(B)**, change in abundance during the three digester phases (P1, P2, P3) which correspond to three stages of succession. Differences in abundance significant at p <0.05.

Whether performance phases represented community successional stages was considered. Multivariate analysis revealed a strong effect of phases (P1–P3) on microbial community composition. The separation among phases observed in both ANOSIM (R = 0.8025, p = 0.0033) and PERMANOVA (F = 18.136, p = 0.0033) indicates that operational phase exerted a dominant influence on microbial community structure. Scatter plots were used to compare total species in the phases. This showed that P2 diverged considerably from P1 (R^2^ = 0.300) while P2 and P3 were quite similar (R^2^ = 0.928), showing again dramatic community turnover (**Figure 2B**). Family level abundance was also used to evaluate successional gradients because these represent a high degree of shared ecological functions (Goldford et al., 2018; Morrissey et al., 2019). Families that peaked during each phase (p < 0.05) included both high and low abundance groups (**Figure 3B**). During P1, the dominant families were *Lactobacillaceae*, *Enterobacteriaceae*, *Corynebacteriaceae* and *Bacillaceae*. The *Enterobacteriaceae*, in particular, was very abundant (18.8%, D1; 26%, D2; 30.3%, D3) but declined dramatically in P2 (0.6 - 1.6%) and P3 (0.6 - 0.9%). The most abundant species in P1 included, *Escherichia coli*, *Enterobacter* sp. 638, *Corynebacterium* sp., and *Salmonella enterica*. These species and families are expected in poultry litter feedstock, and are components of chicken guts (Mei et al., 2017; Shang et al., 2018). Many other families also had highest abundance during phase 1 such as *Brevibacteriaceae*, *Burkholderiaceae*, and *Propionibacteriaceae* (**Supplementary Figure 2**). Dominance during phase 2 (p < 0.05) included families *Synergistaceae*, *Syntrophomonadaceae*, *Eubacteriaceae*, *Peptococcaceae*, *Ruminococcaceae*, *Thermoanaerobacteraceae*, *Lachnospiraceae*, and others (**Figure 3A** and **Supplementary Figure 2**). Family dominance shifted again during phase 3 (p < 0.05) to *Bacteroidaceae*, *Porphyromonadaceae*, *Thermotogaceae*, *Prevotellaceae*, *Flavobacteriaceae*, and others (**Figure 3A** and **Supplementary Figure 2**).

**Figure 4:**
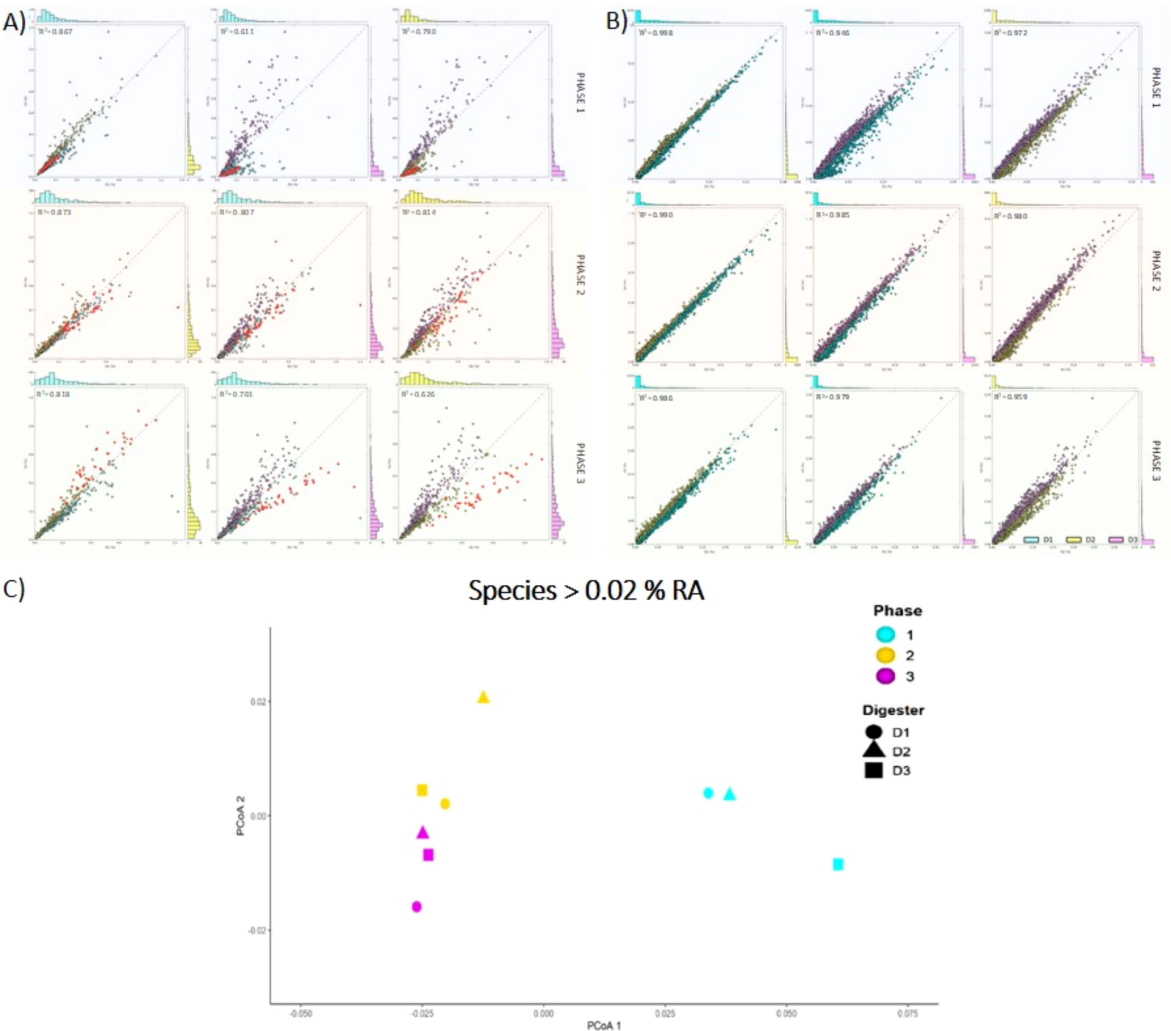
Comparison of similarity of each community successional stage (P1, P2, P3) between digesters using scatter plots: **(A)** species with relative abundance of 0.1 – 0.02% and Bacteroidetes in red, and **(B)** total KEGG functions. **(C)** Bray-Curtis PCoA analysis for species with relative abundance ≥0.02%.

The reproducibility of succession between digesters was further evaluated by comparing corresponding phases using scatter plots. For species with relative abundance >0.1%, D1/D2 had highest similarity during P2 (R^2^ = 0.922); the other comparisons were D1/D3 (R^2^ = 0.887) and D2/D3, R^2^ = 0.809 (**Supplementary Figure 4**). At P3, all three pairings showed decreased similarity, particularly D2/D3 (R^2^ = 0.573). The less abundant species (0.1-0.02%) were also compared: digesters D1 and D2 were most similar at each phase (**Figure 4A**). In addition, the level of similarity increased among all three digesters during P2: D1/D2, R^2^ = 0.873; D1/D3, R^2^ = 0.807; and D2/D3, R^2^ = 0.814 (**Figure 4A**). However, during phase P3, the similarities decreased, and D3 was most divergent: D1/D2, R^2^ = 0.818; D1/D3, R^2^ = 0.701; D2/D3, R^2^ = 0.626 (**Figure 2**). Beta diversity was measured for species >0.02% using Bray-Curtis dissimilarity and PCoA. This also showed three successional stages (**Figure 4C**).

**Figure 5:**
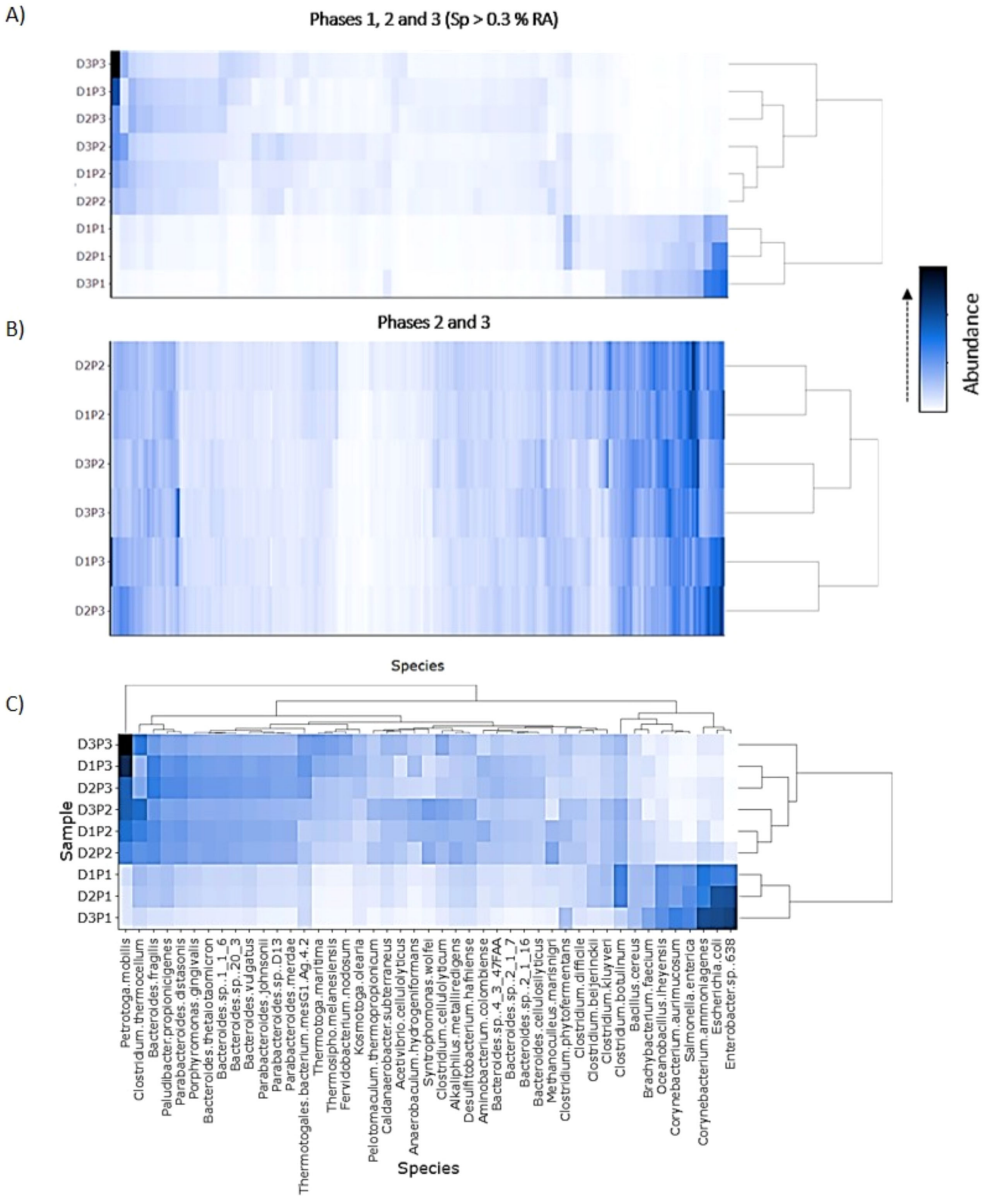
Hierarchical clustering showing the relative abundance of species: **(A)** ≥0.3% for all digesters (D1, D2, D3) during all phases (P1, P2, P3); **(B)** 0.1-0.02% for P2 and P3; and **(C)** the 45 most abundant species.

Hierarchical cluster analysis was used to evaluate the trajectories of community structure across the successional gradient. This was done by subdividing species into four abundance groups. First, the top 45 most abundant species were considered. The three phases clustered separately and D1/D2 were most similar (**Figure 5C**). The majority of the abundant species increased during phase 2, including nine species of *Bacteroides* and five *Clostridium* species. Second, all species with relative abundances of >0.3% (75 spp.) (**Figure 5A**) and >0.2% (121 spp.) (**Supplementary Figure 5**) were compared across all phases. In each case, phases clustered separately and D1/D2 had highest similarity. The most divergent sample was D3 in phase P3. Third, low abundance species (0.1-0.02%) also showed succession: D1 and D2 were more similar to each other than to other phases of the same digester, and, once again, D3 remained distinct in phases 2 and 3 (**Figure 5B**). Clustering was also used to compare species abundance profiles of several dominant phyla (Firmicutes, Bacteroidetes, Euryarchaeota): D1/D2 showed greatest similarity through all three phases (**Supplementary Figure 6**). In summary, reproducible successional dynamics were found for both high and low abundance populations, a dramatic shift in species abundances occurred as the methanogenic food web formed, and two digesters were consistently most similar (D1/D2) suggesting two successional pathways might be present.

**Figure 6:**
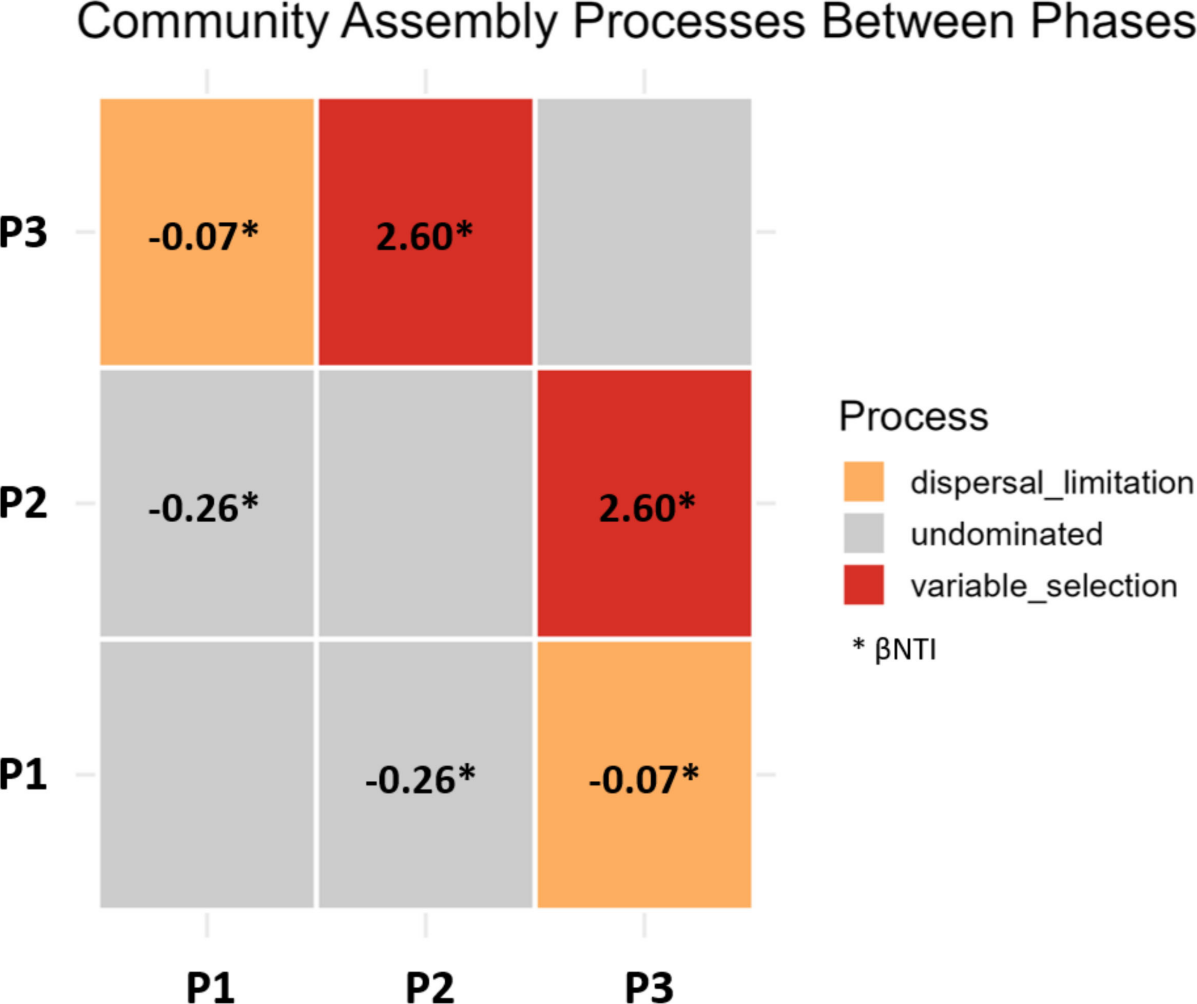
Tests of stochastic and deterministic mechanisms during secondary succession in three digesters. Pairwise comparisons were made for the three operational phases (P1, P2, P3) using the digesters as replicates. βNTI values are reported in the boxes. RCbray analysis was used to determine whether dispersal or selection were dominant processes.

To estimate the ecological processes involved in secondary succession we used modeling with βNTI analysis (Stegen et al., 2012). This method determines whether the composition of communities can be associated with random (stochastic) or deterministic processes. If the |βNTI| > 2, deterministic processes are dominant and responsible for the differences between pairwise comparisons of communities. But if the |βNTI| < 2, stochastic processes are dominant (Stegen et al., 2013). In addition, the RCbray metric was used to assess the contributions of drift and dispersal. The three digester phases were compared to each other (**Figure 6**). The transition from P2 to P3 was classified as deterministic with a βNTI value of 2.60. The other comparisons (P1/P2, P1/P3) had values close to zero indicating a greater contribution of stochastic processes. Following Stegen et al. (2015), the classification of the community assembly processes for each of these transitions is undominated (P1 → P2), variable selection (P2 → P3), and dispersal limitation (P1 → P3) (**Figure 6**).

### 3.3 Succession of community functions in replicate digesters

**Figure 7:**
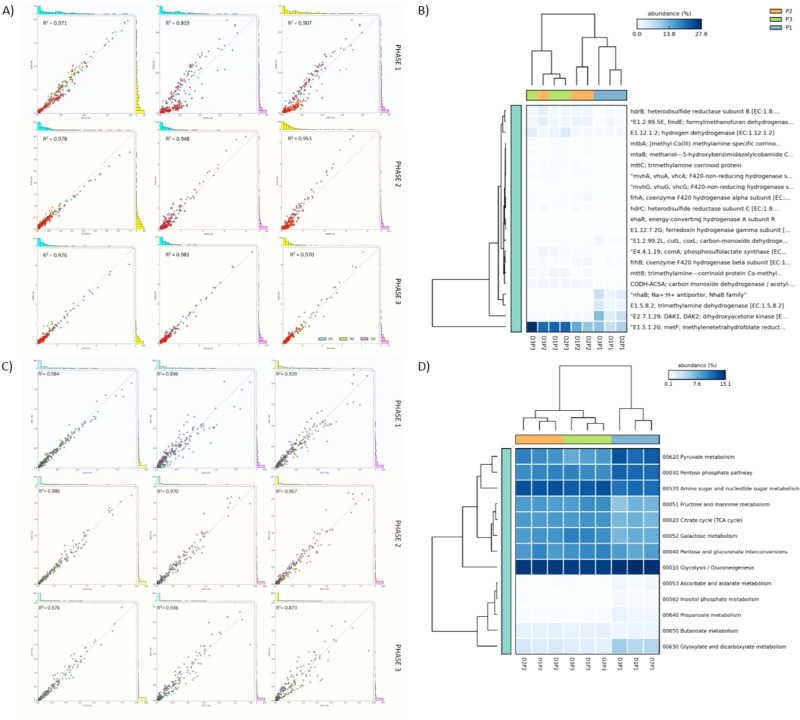
Scatter plots of KEGG energy **(A)** and carbohydrate **(C)** functions during the three successional stages (P1, P2, P3). Methane metabolism functions are highlighted in red. Cluster analysis of KEGG methanogenesis functions **(B)** and carbohydrate functions **(D)**.

First, we tested whether performance phases represented successional stages. Total functions in P1 compared to P2 showed correlation of R^2^ = 0.814, while P2 versus P3 was higher (R^2^ = 0.983) (**Figure 2B**). This indicates increasing similarity over time. Combined energy functions from all digesters also showed low correlation between P1 and P2 (R^2^ = 0.630) but much higher correlation for P2 versus P3 (R^2^ = 0.978) (**Figure 2B**). The same pattern was found for combined carbohydrate functions (P1/P2, R^2^ = 0.697; P2/P3, R^2^ = 0.969) (**Figure 2B**). Second, to evaluate the reproducibility of successional stages regarding functions, each phase was compared to the corresponding phase in the other digesters. Combined functions showed very high correlations between equivalent phases although D1/D2 was somewhat higher (**Figure 4B**). Correlations between digesters dropped slightly during P3 (**Figure 4B**). In addition, two KEGG subsystems were evaluated separately. Comparing total energy functions showed that D1 and D2 were more similar to each other than to D3 during the first two phases (**Figure 7A**). However, D3 increased in similarity during phase 3. Total carbohydrate functions also had highest correlation between D1/D2 (**Figure 7C**). In contrast to energy functions, though, carbohydrate function similarity decreased in the final phase (P3), particularly in D3 (**Figure 7C**). Cluster analysis of carbohydrate and methanogenesis functions also showed clear discrimination between the trajectories of D1/D2 and D3 (**Figures 7B, D**).

Specific examples of energy and carbohydrate metabolism, and cell physiology, were also evaluated separately. Similar to community structure, some KEGG functions peaked (p < 0.05) during each phase. Examples are: phase 1, nitrogen, fatty acid, pyruvate and propanoate metabolisms, phosphotransferase system, and polycyclic aromatic hydrocarbon (PAH) metabolism; phase 2, acetate metabolism (acetate kinase [*ackA*] and phosphate acetyltransferase [*pta*] genes) and butanoate metabolism, aminoacyl-tRNA synthetase, ribosomes, and carbon fixation; and phase 3, TCA cycle, glycan degradation, and galactose/fructose/mannose metabolisms (**Figure 3B**, **Supplementary Figure 3**).

## 4 Discussion

We evaluated whether the start-up of batch digesters exhibited repeatable assembly pathways for community structure and function when a pre-adapted methanogenic microbiome was disassembled (diluted) and placed into a similar environment, but with a resource gradient and hundreds to thousands of foreign species. All three replicates exhibited directional change along the gradient which was created by feedbacks between microbiome metabolism and resource quality and quantity. Highly reproducible stages of secondary succession were found that corresponded to three phases of digester performance: (1) methane inhibition (start-up), (2) high methane, and (3) low methane plateau. Each stage was characterized by differences in community structure, including peaks in abundance of particular species and families. We also found successional dynamics for energy and carbohydrate metabolisms and cell physiology. With about 1600 identified species in each digester, the shifts in species and functions during succession were surprisingly similar between digesters. The balance between stochastic and deterministic processes was also found to shift along the successional gradient.

### 4.1 Stages of secondary succession along a resource gradient

The well-known methanogenic food web contains the steps hydrolysis, acidogenesis, acetogenesis, and methanogenesis. Since methane production requires an integrated food web, both species and functions are expected to reflect successional states. In stage 1 (early succession), the severe dilution of the methanogenic community should disrupt positive interspecific interactions. As expected, methane and total biogas production were temporarily inhibited. However, the restart of methane production did occur fairly quickly indicating recovery of these interactions. To provide H_2_, CO_2_ and acetate for methanogenesis, hydrolysis and fermentation of the most labile substrates (sugars, amino acids) are expected to occur first (Pascual-García and Bell, 2020). Supporting this inference, peaks occurred in the phosphotransferase system which transports sugars across membranes (Somavanshi et al., 2016). Propionate levels and propanoate pathway genes were also highest.

Stage 2 (mid-succession) was characterized by peak biogas and methane production and the greatest changes in community structure and function. The resource gradient changed as total organic carbon greatly decreased and food web intermediates (acetate, CO_2_) increased. This was reflected by a shift in community functions, particularly energy and carbohydrate metabolism. Strong positive interspecific interactions, and high nutrient levels, are expected to underlie maximum methane production (Embree et al., 2015). Evidence for mutualistic interactions involving syntrophic acetate-oxidizing bacteria are peaks in acetate levels, acetate metabolism genes (*ackA*, *pta*), and hydrogenotrophic methanogens (*Methanomicrobiaceae)* (Hattori, 2008; Li et al., 2022). Families containing syntrophic bacteria, *Syntrophomonadaceae*, *Synergistaceae* and *Thermoanaerobacteraceae*, did peak at this time (Zhu et al., 2019). Recolonization of the disturbed environment was further indicated by peaks in ribosome and aminoacyl tRNA biosynthesis genes which represent cell growth (Nemergut et al., 2015). A dramatic change in dominant and minor species also occurred, including the highest convergence among digesters. This was not necessarily expected because of the dilution and high number of foreign species that could potentially invade (Amor et al., 2020; Bittleston et al., 2020). The convergence of minor populations is particularly noteworthy and may represent prior environmental filtering of niche specialists. For example, using replicated freshwater microcosms, Pagaling et al (Pagaling et al., 2014). found greater predictability of assembly when the community was “pre-conditioned” by prior exposure to a particular environment.

Late succession (stage 3) showed the greatest convergence in performance properties. Levels of propionate, butyrate, acetate, COD and pH were most similar among all digesters. Both methane and total biogas substantially decreased, indicating a large decline in readily digestible organic compounds, as well as the lowest levels of propionate and acetate. The original labile substrates are expected to be depleted as decomposition shifts toward recalcitrant organic matter such as cellulose (Rivett et al., 2016). Supporting this hypothesis, carbohydrate functions showed peaks for glycan degradation and metabolism of galactose/mannose which are components of hemicellulose. TCA cycle genes increased while pyruvate metabolism genes decreased indicating shifts from fermentation to respiration. Although high free ammonia can inhibit methanogens, the values here correspond to increased methane production, suggesting that inhibition was minimal or that ammonia-tolerant methanogenic populations were active (Yang et al., 2025). Families peaking included *Thermotogaceae*, *Porphyromonadaceae* and *Bacteroidaceae* which produce enzymes for hydrolysis, as well as polysaccharide catabolism, including cellulose and hemicellulose (Hahnke et al., 2015; Ozbayram et al., 2018; Esercizio et al., 2021). Due to the large reduction of methane and acetate, we predict decreases in strong mutualistic interactions which is consistent with reductions in syntrophic families.

### 4.2 Stochasticity, determinism, and contingency

We found evidence for a shift in the balance of stochastic and deterministic mechanisms during secondary succession. Using the conceptual system of Stegen et al (Stegen et al., 2015), the transition from stage 1 to 2 can be classified as the undominated process where neither dispersal nor selection were most important. This was likely due to the initial mixing of inoculum and liquid feedstock which created random dispersal in the reactors, followed by community convergence when the methanogenic food web was reassembled. In contrast, the transition from stage 2 to 3 was classified as deterministic with variable selection. We propose that variable selection represented an increase of environmental heterogeneity even though the digester had a liquid environment. This could be due to the slow, irregular breakdown of plant biomass and particulate organic matter in the feedstock (Allison, 2012; Enke et al., 2018).

The increase of deterministic selection during the stage 2–3 transition was associated with divergence of minor populations and carbohydrate functions. We suggest these minor species are specialist degraders of the recalcitrant complex organic matter (Rivett et al., 2016) and utilize carbohydrate-active enzymes (CAZymes) (Berlemont and Martiny, 2016). This is consistent with the expected increase in the heterogeneity of debris biomass during decomposition (Enke et al., 2018). Yu et al., 2024 also found that species with specialized functions can coexist when they share a major resource. These factors may explain why Bacteroidetes, which are specialized polysaccharide degraders (Bertucci et al., 2019), converged in abundance during the initial consumption of labile substrates such as sugars, then diverged during later succession. In addition, the particulate material might have become progressively colonized during the long period of digestion which would further increase environmental variability (Enke et al., 2018). Other possible reasons for the divergence of minor populations could be that the same species have different dynamics in different contexts (Bittleston et al., 2020), or that species interactions (mutualistic versus competitive) changed as nutrients varied (Hoek et al., 2016). Therefore, we propose that the highest degree of determinism was associated with variable selection of minor populations responding to heterogeneous resource quality and spatial complexity.

Deterministic assembly has been indicated in other digester studies as well. For example, Vanwonterghem et al., 2014. found highly parallel changes in community succession in replicate CSTR digesters. However, this study and others (Peces et al., 2018) used quite different experimental conditions, particularly synthetic feedstock. Niche specialization was found by Huber et al., (2020). in a stressed thermophilic digester treating poultry litter when performance recovery coincided with recovery of a specific population rather than replacement by a redundant species. In our study, the parent digester received poultry litter feedstock for more than three years which could facilitate development of mutualistic interactions. The convergence of minor populations also suggests the development of synergistic networks (Nobu et al., 2015; Wu et al., 2016). Previous work with our digesters has shown changes in network organization and modularity during succession which may indicate niche specialists and mutualism (Guerrero-Toledo, 2019). A time-scale dependence for assembly mechanisms was also found. When early succession (P1) was compared to late succession (P3), dispersal limitation was indicated, likely due to the closed nature of the reactors where no new species could immigrate from a regional species pool. This shows that the time scale for sampling successional gradients is important for discerning assembly processes (Dini-Andreote et al., 2015).

Our study also found preliminary evidence for the effects of historical contingency on successional pathways (Fukami, 2015). Although similar successional stages were present in all three digesters, species and functions could be distinguished as two trajectories (D1/D2 and D3) even with the same set of species. For dominant and minor species, as well as functions, the two digesters with the highest initial similarity (D1, D2) showed the most similar successional pathways. In contrast, D3 was less similar and showed delayed production of methane. The difference of D3 at stage 1 was undoubtedly due to a sampling effect. Even though all three digesters were started by equally dividing the inoculum-feedstock mix, D3 showed the highest initial variation in populations. Pascual-Garcia et al (Pascual-García et al., 2025). found that small differences in community composition can lead to different successional trajectories. In our study, the majority of bacteria and methanogens that became dominant in mid and late succession of D1/D2 began in lower abundance in D3 and never reached similar levels. For example, species associated with plant biomass degradation (*Clostridium cellulolyticum*, *C. phytofermentans*, *C. thermocellum*) increased slowly in D3. However, it is important to note that clustering is a descriptive statistic that requires further confirmation, ideally with additional replicate reactors, but this was not feasible given the size and cost of our experiment.

### 4.3 Limitations and applications

Several limitations and applications of our study can be mentioned. First, an important point is that the disturbed digester microbiomes were already pre-assembled following long-term selection which provided prior opportunity for competitive exclusion and invasion from the feedstock metacommunity. Whether highly reproducible succession would occur with entirely different feedstock is a relevant question. A complementary experiment could use another feedstock with new foreign species and evaluate whether different alternative successional pathways arise. We further speculated that environmental heterogeneity increased in late succession due to progressive decomposition and colonization of organic debris. This hypothesis can be tested by isolating organic particulate matter over time and measuring diversity. Whether alternative transient states are an important feature of AD also deserves further scrutiny. Indeed, one strength of anaerobic digestion is the adaptability of the microbiome toward operational changes suggesting significant breadth in operational community states; nevertheless, normal operation can occasionally slide into dysfunction for no apparent reason. Regarding industrial applications, this study can contribute to defining “microbiome design principles” where controlling bioreactor community assembly is a fundamental objective of the ecological engineering approach (Lawson et al., 2019). Finally, how these findings transfer to steady state digesters remains to be tested. It may be that the undominated mechanism for community assembly recurs with every pulse of fresh feedstock. A deeper understanding of community assembly processes in anaerobic digestion may help to engineer resilience, product quality, and new applications.

## Data Availability

The original contributions presented in the study are publicly available. This data can be found here: NCBI SRA, accession PRJNA1327443.
