# Supplementary material for: Community assembly following disturbance in batch anaerobic digesters displays highly reproducible secondary succession and a shifting stochastic-deterministic balance
Source: Front Microbiomes. 2026 Jan 27;4:1707779. doi: 10.3389/frmbi.2025.1707779 (PMC12993682; doi:10.3389/frmbi.2025.1707779)
Supplement: Supplementary file 1 [file DataSheet1.pdf]

Community assembly pathways following disturbance in batch anaerobic digesters display determinism, contingency and alternative states

Flor de Maria Guerrero-Toledo[1,2,3], Teodoro Espinosa-Solares[1], Guadalupe Hernandez-Eugenio[1], David H. Huber[2,3]

Departmento de Ingeniería Agroindustrial, Universidad Autónoma Chapingo, México[1]

Department of Biology[2] and Gus R. Douglass Institute[3], West Virginia State University, Institute, West Virginia, 25112

Supplementary Figures (1 – 6)

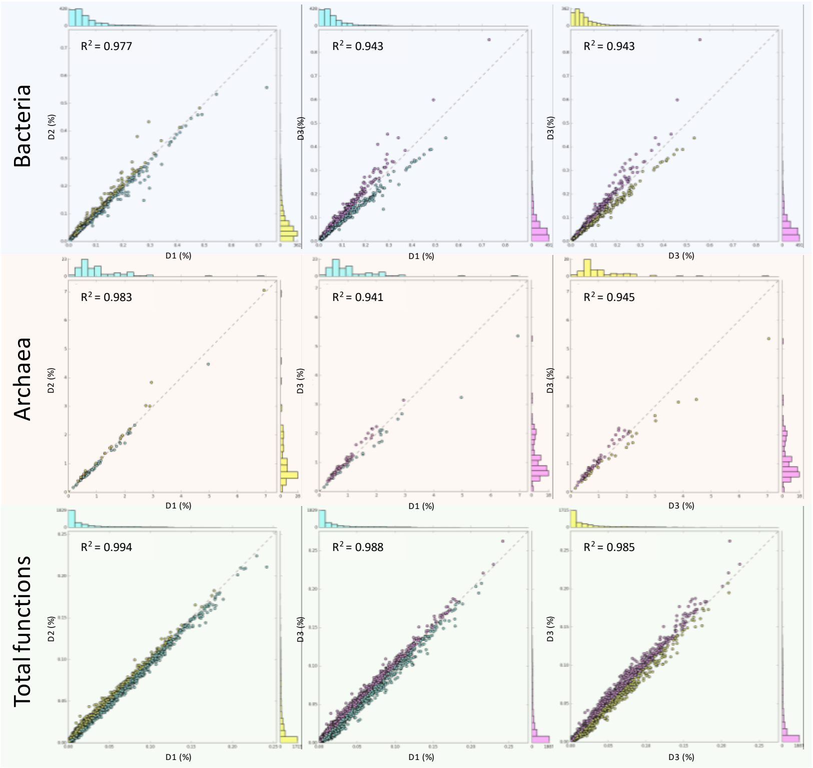

Figure 1S. Scatter plots comparing the three digesters (D1, D2, D3) as replicates. The three rows of plots represent comparisons of total species of bacteria, archaea, and KEGG functions.

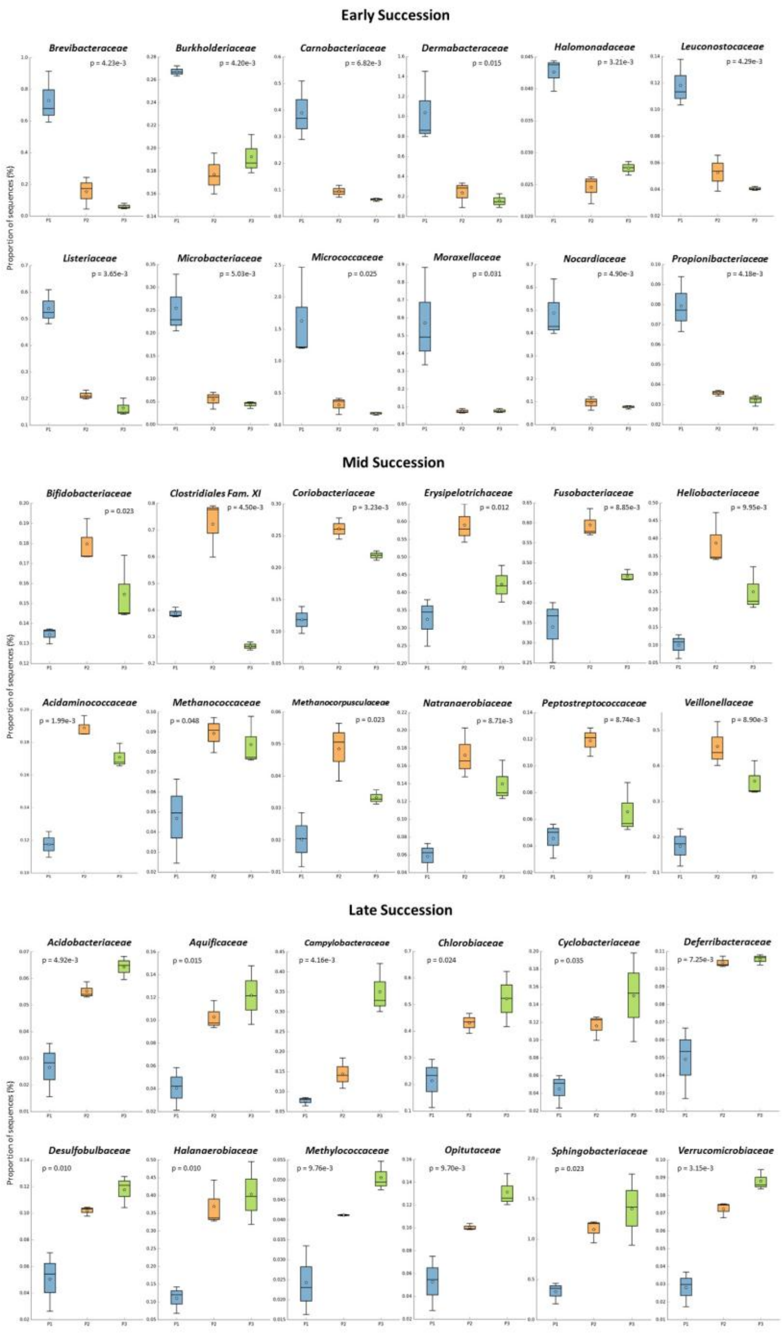

Figure 2S. Box plot analysis showing that particular bacterial families peak in abundance during each of the three digester performance phases (P1, P2, P3) which corresponds to three stages of secondary succession (early, mid, late). Differences in abundance significant at $p < 0.05$.

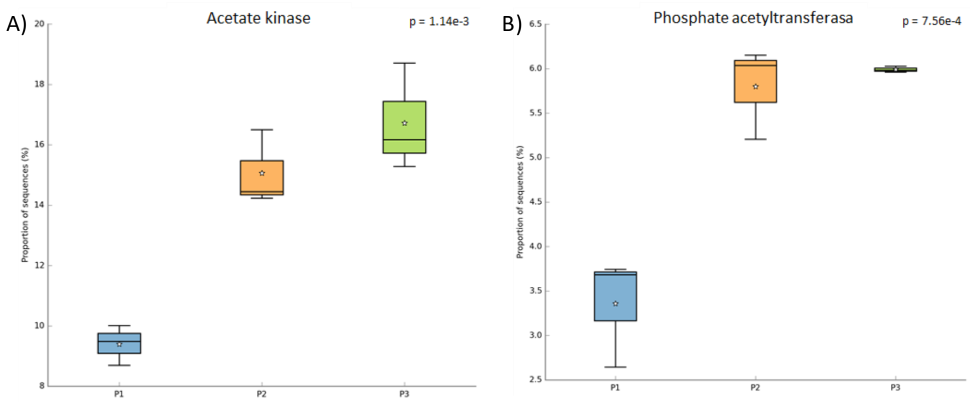

Figure 3S. Box plots showing change in abundance for acetate metabolism genes (A) acetate kinase (*ackA*), and (B) phosphate acetyltransferase (*pta*). Differences in abundance at $p < 0.05$.

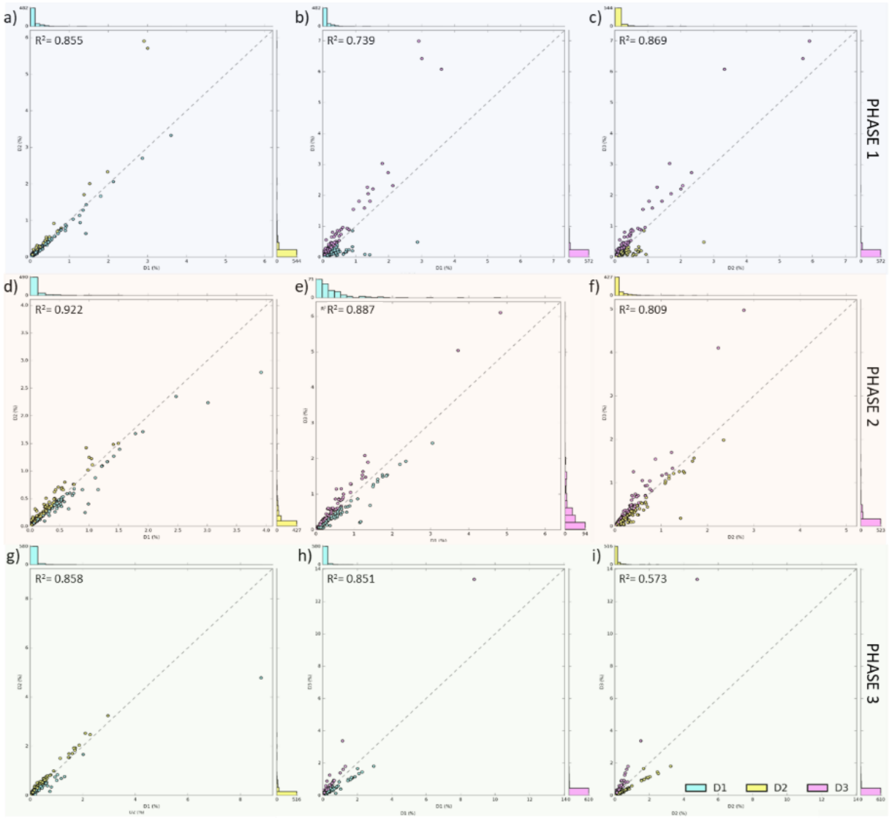

Figure 4S. Comparison of similarity of each community successional stage (performance phases P1, P2, P3) between digesters using scatter plots. Species have relative abundances ≥0.1%.

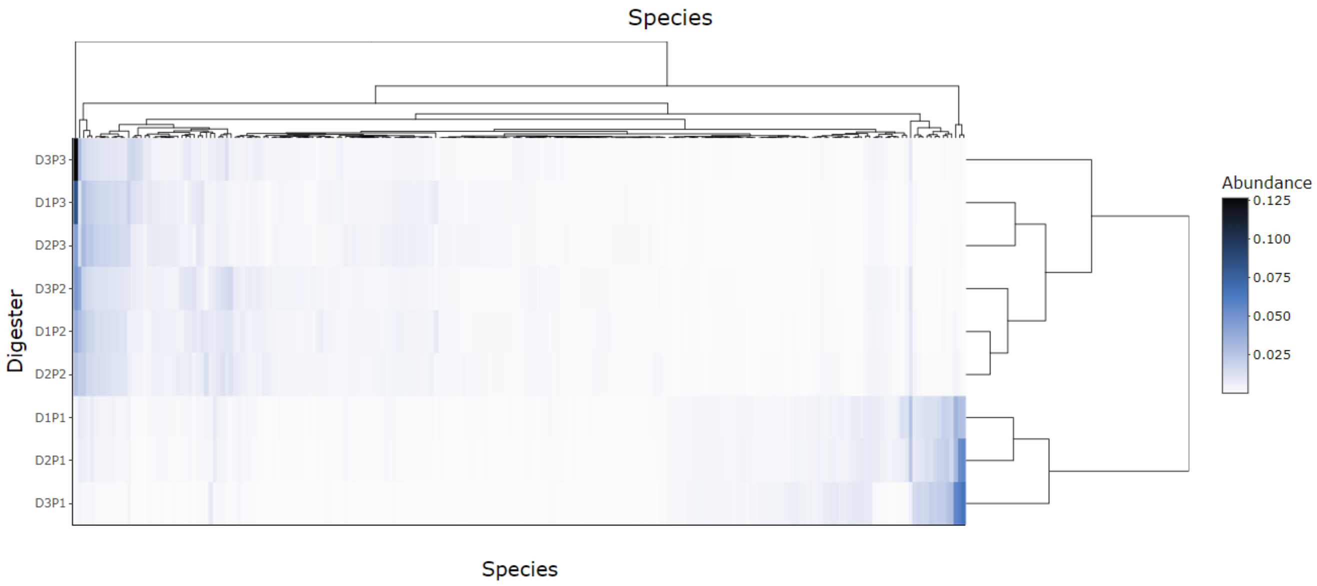

Figure 5S. Hierarchical cluster analysis for all species ≥0.02% relative abundance. Digesters (D1, D2, D3). Phases (P1, P2, P3).

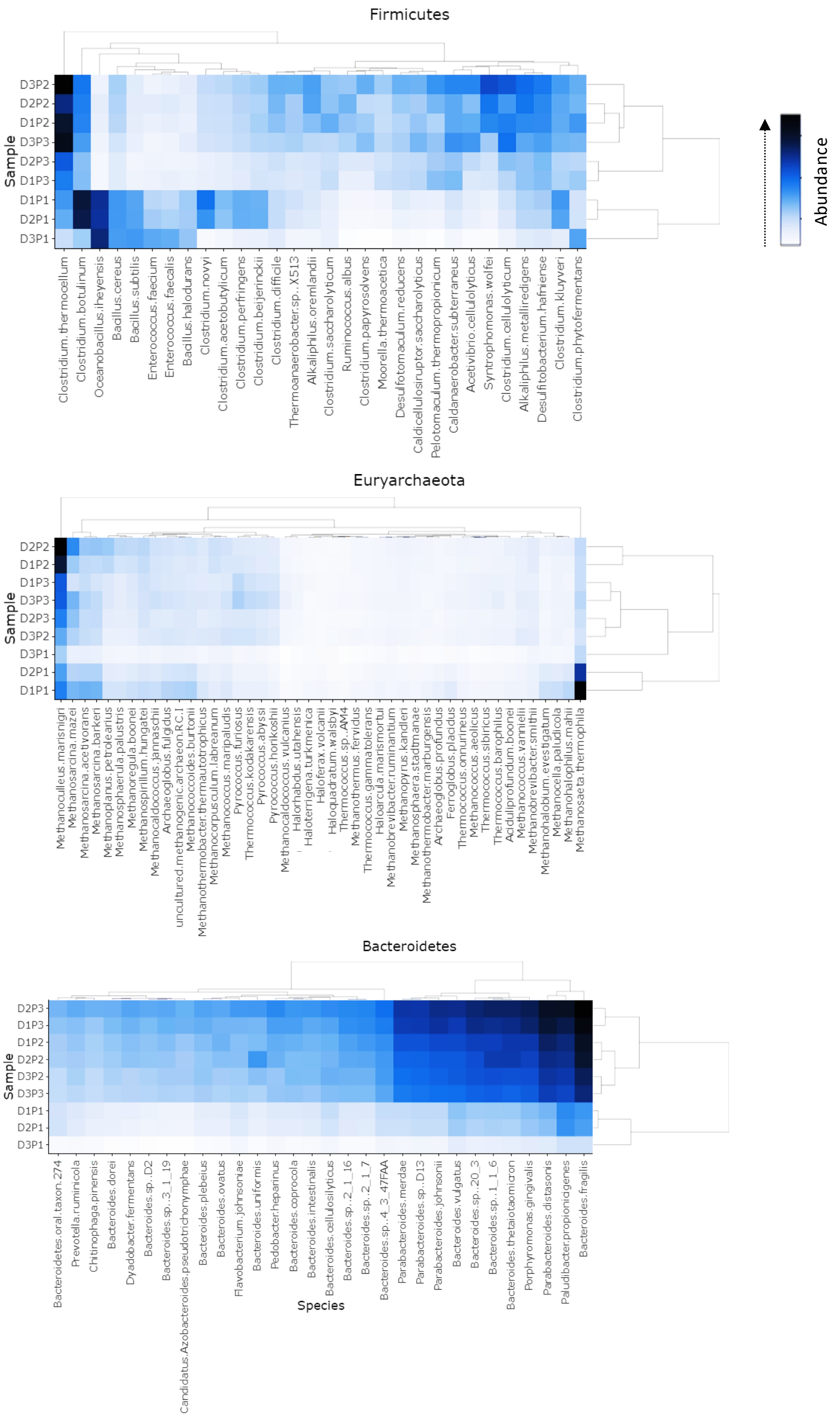

Figure 6S. Hierarchical cluster analysis showing the relative abundance of species for three abundant phyla, Firmicutes, Bacteroidetes, and Euryarchaeota.
